# Infinity war: *Trichomonas vaginalis* and interactions with host immune response

**DOI:** 10.15698/mic2023.05.796

**Published:** 2023-03-30

**Authors:** Giulia Bongiorni Galego, Tiana Tasca

**Affiliations:** 1 Grupo de Pesquisa em Tricomonas, Faculdade de Farmácia e Centro de Biotecnologia, Universidade Federal do Rio Grande do Sul, Avenida Ipiranga, 2752, Porto Alegre, 90610-000, Rio Grande do Sul, Brazil.

**Keywords:** trichomoniasis, Trichomonas vaginalis, immune response, inflammation

## Abstract

*Trichomonas vaginalis* is the pathological agent of human trichomoniasis. The incidence is 156 million cases worldwide. Due to the increasing resistance of isolates to approved drugs and clinical complications that include increased risk in the acquisition and transmission of HIV, cervical and prostate cancer, and adverse outcomes during pregnancy, increasing our understanding of the pathogen's interaction with the host immune response is essential. Production of cytokines and cells of innate immunity: Neutrophils and macrophages are the main cells involved in the fight against the parasite, while IL-8, IL-6 and TNF-α are the most produced cytokines in response to this infection. Clinical complications: *T. vaginalis* increases the acquisition of HIV, stimulates the invasiveness and growth of prostate cells, and generates an inflammatory environment that may lead to preterm birth. Endosymbiosis: *Mycoplasma hominis* increased cytotoxicity, growth, and survival rate of the parasite. Purinergic signaling: NTPD-ases and ecto-5'-nucleotidase helps in parasite survival by modulating the nucleotides levels in the microenvironment. Antibodies: IgG was detected in serum samples of rodents infected with isolates from symptomatic patients as well as patients with symptoms. However, antibody production does not protect against a reinfection. Vaccine candidate targets: The transient receptor potential- like channel of *T. vaginalis* (TvTRPV), cysteine peptidase, and α-actinin are currently cited as candidate targets for vaccine development. In this context, the understanding of mechanisms involved in the host-*T. vaginalis* interaction that elicit the immune response may contribute to the development of new targets to combat trichomoniasis.

## INTRODUCTION

The unicellular protozoan *Trichomonas vaginalis* is the etiologic agent of human trichomoniasis, the most common non-viral sexually transmitted infection (STI) in the world. The figures of incidence are higher than *Chlamydia* infection, gonorrhea, and syphilis. In 2016, the World Health Organization (WHO) estimated an incidence of 156 million cases worldwide, while in the United States of America, according to the Center for Disease Control and Prevention (CDC), there was an incidence of 6.9 million in 2018 with direct medical costs of $144 million [1, 2]. Both men and women can be asymptomatic. In women, when symptoms occur, they manifest by vaginal discharge, itching, dysuria, and abdominal pain. In men, the most common symptoms are prostatitis, decreased sperm motility, and epididymitis [3]. In addition, several studies have shown that *T. vaginalis* infection may increase the acquisition and transmission of HIV in women [3, 4]. Co-infection with *Chlamydia*, gonorrhea, syphilis, and herpes simplex virus type 1 and 2 have also been described [3].

Currently, there are three drugs approved by the Food and Drug Administration (FDA) against trichomoniasis: metronidazole, tinidazole, and secnidazole, representatives of the 5-nitroimidazole class [1, 5]. However, the rate (11 to 28%) of metronidazole-resistant isolates is an increasing problem [5]. The first metronidazole-resistant isolate was detected in 1962, two years after its introduction [6]. Likewise, the newest therapeutic alternative, secnidazole, has already resistant isolates [5]. Furthermore, the tests with the highest sensitivity and specificity for detection of *T. vaginalis*, known as nucleic acid amplification tests (NAATs), are not routine in most of clinical laboratories. This, together with the fact that trichomoniasis is not a reportable STI, may contribute to underdiagnosis and consequently underestimation of the number of cases [7].

In this context, the understanding of the mechanisms involved in the interaction between *T. vaginalis* and the host immune response may contribute to the development of new targets to fight the parasite. The search strategy here was based on the combination of *T. vaginalis* or trichomoniasis with different keywords: immune, immunity, macrophages, neutrophils, immune response, innate immunity, adaptive immunity, purinergic signaling, pregnancy, antibody, *Mycoplasma hominis*, vaccine, and HIV. Only articles indexed in the PubMed Central^®^ database, in English or Portuguese, published between 2000 and 2022 were included in this review in order to summarize the updated current data on the trichomoniasis immune response field.

## THE AVENGERS: THE CELLS OF THE HOST IMMUNE SYSTEM AND CYTOKINE PRODUCTION

During an infection, host defense begins with innate immunity mediated by myeloid cells, natural killer cells, innate lymphoid cells, the complement system, and defensins [8]. Neutrophils are polymorphonuclear cells (PMNs), one of the most important members of the immune response. They can be recruited by pattern recognition receptors present on endothelial cells or inflammatory mediators released by resident leukocytes in tissues [9].

Recently, analyses of *T. vaginalis* lipophosphoglycan (LPG) concluded that the glycan core lacks significant amounts of phosphate compared to other LPGs, such as *Leishmania major* LPG. Thus, changing the term *T. vaginalis* LPG for *T. vaginalis* lipoglycan (TvLG) is suggested. The poly-LacNAc/LNB chains present in TvLG seem to be required for the parasite to bind to host ectocervical cells [10]. Human ectocervical and endocervical cells, as well as human vaginal epithelial cells (VECs) produced IL-8 and macrophage inflammatory protein (MIP)-3α in response to TvLG. IL-8 is associated with neutrophil migration to the infection site, while MIP-3α is a chemokine that attracts immune cells and is also required for the maturation of dendritic cells [11]. VECs, in the presence of live *T. vaginalis*, increased IL-6 and IL-8 production, as well as monocyte chemoattractant protein-1 (MCP-1) mRNA expression. As a consequence, the media collected from culture supernatants of VECs in contact with *T. vaginalis* (TCM) increased the migratory ability of neutrophils and mast cells. In turn, supernatants of mast cells incubated with TCM also stimulated neutrophil migration [12]. Interestingly, the species of bacterial vaginosis (BV) prevalent at the site of infection at the time of parasite inoculation seem to be related to the immunological effects exerted by the parasite. For example, *Prevotella bivia*, a common agent of bacterial vaginosis, increased IL-8 production induced by the protozoan, whereas MIP-3α induction was lower when both microorganisms were together vs. *P. bivia* alone. On the other hand, *T. vaginalis* decreased *Lactobacillus* species typically present in the vaginal microbiota of women without BV [13]. IL-8 is a proinflammatory cytokine which can regulate the activation, migration, and degranulation of neutrophils. It is released with inflammatory stimulations. Adhesion or contact between the parasite and neutrophils is essential for the production of this cytokine; the membrane integrity of the protozoan plays a role in this too since lysates of trichomonads and *T. vaginalis* secretory products (TvSP) are not able to produce the same amount of IL-8. The pathways that likely contribute to promoting increased cytokine transcription are the nuclear kappa B (NF-kB) and mitogen-activated protein kinase (MAPK) [14]. Although secretory products do not stimulate neutrophils in the same way as live trichomonads do, they also contribute to IL-8 production possibly due to lipid mediator leukotrienes (LTB_4_) in their composition. It is hypothesized that LTB_4_ interacts with high affinity (BLT1) and low affinity (BLT2) LTB4 receptors present on neutrophils and activate the NF-kB and cAMP response element-binding (CREB) pathway. Both appear to be involved in cytokine production [15, 16]. Human vaginal and cervical epithelial cells express and secrete galectin-1 and galectin-3, which play opposite roles during trichomoniasis and act as pathogen recognition receptors for TvLG. In this context, galectin-1 has immunosuppressive properties, while galectin-3 has immunostimulant ones. *T. vaginalis* decreased galectin-3 levels, which decreased the LG pattern recognition. As a result, there is an expected decrease in the recruitment of macrophages and neutrophils as this galectin mediates infection clearance. Meanwhile, the parasite can benefit from the high binding affinity of TvLG to galectin-1, which decreases chemokines levels, such as IL-8 and MIP-3α. IL-8 is expected to reduce the recruitment of neutrophils and macrophages, while MIP-3α is expected to reduce dendritic cells [17].

The pathogen-neutrophil interaction goes beyond cytokine production. This *in vitro* direct contact decreases the myeloid cell leukemia 1 (Mcl-1) expression, an anti-apoptotic protein previously associated with PMNs survival, and increases caspase-3 expression, a protein related to spontaneous apoptosis in neutrophils that triggers the acceleration of the apoptosis process [18]. Interestingly, while live trophozoites enhanced the rate of neutrophil apoptosis, trichomonads lysate reduced it. *T. vaginalis* lysate co-incubation with PMNs maintained Mcl-1 expression for a longer time than the control group did. In addition, there was a reduction in caspase-3 activation after 15 and 25 hours of incubation, while caspase-3 cleavage by the untreated group occurred after 15 hours of incubation [19]. Reactive oxygen species (ROS) function as signaling messengers. Increased ROS induces mitogen activated protein kinases, protein kinase C, and stimulation of cell proliferation or death. In neutrophils, ROS production aids in the elimination of ingested pathogens. In response to the presence of trichomonads, neutrophils increase intracellular ROS via the NADPH oxidase system, resulting in a possible caspase-3 activation. Activation of caspase-3 has been suggested as part of the apoptosis pathway induced by *T. vaginalis* in human neutrophils [20]. Trichomonad lysate, secretory product, and membrane components stimulated superoxide anion production by neutrophils as a possible host defense mechanism. In contrast, this production was lower due to protozoan peptidase inhibition [21]. Inhibition of adenosine deaminase, the enzyme that converts adenosine into inosine, leads to increased adenosine bioavailability. As a result, there are low ROS and IL-8 levels due to the anti-inflammatory effects of adenosine [22]. Meanwhile, nitric oxide (NO) is also involved in immune defense mechanisms, and its production by neutrophils has been reported. In the presence of inosine and adenosine, a nucleoside with an immunosuppressive effect, there is a decrease in NO generation by PMNs, suggesting a contribution to the establishment of infection [23]. Furthermore, the parasite could degrade NO in anaerobic conditions probably due to the action of A-type flavoprotein, an NADH-dependent enzyme that responds to NO species exposure. During nutrient deprivation, overexpressed *T. vaginalis* macrophage migration inhibitory factor (TvMIF) was detected, which is a human cytokine homolog that mimics the HuMIF (human macrophage migration inhibitory factor). It has been proposed that this overexpression of TvMIF can inhibit ROS production and prevent ROS-induced apoptosis [24, 25]. Interestingly, reactive nitrogen intermediates (RNI) may be associated with establishing an infection. In order to eliminate an infection, macrophages can produce RNI in response to increased production of proinflammatory cytokines. Isolates of *T. vaginalis* from symptomatic patients inoculated into mice generated higher levels of RNI in vaginal tissue than isolates from asymptomatic ones. This was a possible way to decrease protozoa population in vaginal washings. In contrast, leukocytes showed a higher RNI concentration and nitric oxide isoform (iNOS) protein band intensity in the asymptomatic group. Meanwhile, RNI in vaginal washes and plasma were higher in the asymptomatic group [26, 27].

VECs and human prostatic epithelial cells in contact with *T. vaginalis* or rTvα-actinin 2 increased the production of IL-10, IL-12, IL-6, and tumor necrosis factor alpha (TNF-α). Meanwhile, co-incubation of bone marrow-derived dendritic cells (BMDCs) with TvLG increased the dendritic cell activation markers CD80 and CD86 and the major histocompatibility complex (MHC), opposed to MHC-II expression, which decreased after rTvα-actinin, an immunogenic protein found in the parasite's secretome. Likewise, BMDCs co-incubated with TvLG and later with TvSP decreased the expression of MHC-II. Nevertheless, when TvSP was present, even with prior TvLG stimulation, IL-10 production increased and IL-12 decreased. TvLG-stimulated BMDCs were subsequently treated with rTvα-actinin 2. Inducible regulatory T cells (iTreg) were incubated with these cells. As a result, iTregs showed a greater release of IL-10, TGF-β, and IFN-γ. These data suggest that rTvα-actinin 2 may act as an immunomodulator [28, 29].

Macrophages are also members of the innate immune system and play a crucial role in the host defense against pathogens. These cells can secrete cytokines, defensins, ROS, and NO [30]. Thus, the influence of macrophages during *T. vaginalis* infection was also explored. *In vitro*, human macrophages in the presence of apoptotic neutrophils induced by *T. vaginalis* increased IL-10 production and decreased IL-6 and TNF-α [31]. However, in the presence of lysate, opsonized or live trichomonads, human macrophages increased IL-1β, IL-6, and TNF-α levels. The latter may be dependent on NF-kB activation by NO [32]. *T. vaginalis* adhesion to macrophages is associated with cytokine production. In the first minutes of the adhesion process, there was degradation of IkB-α, likely leading to a reduction in NF-kB activation since IkB-α prevents the binding of NF-kB. However, in a longer adhesion time (eight hours), there was inhibition of NF-kB and consequently a reduction in the TNF-α and IL-12 production. This suggests a possible pathway that parasites use to avoid the generation of cytokine [33]. In mouse macrophages, cytokine production associated with the toll-like receptor (TLR)-2. The presence of the parasite causes phosphorylation of NF-kB p65, p38, and ERK via TLR-2. In addition, p38 and ERK activation resulted in an upregulated expression of the proinflammatory cytokines IL-6, TNF-α and INF-γ, while NF-kB translocation to nucleus via TLR-2 is suggested as an activation pathway. NF-kB activation has been associated with the regulation of NO production [34].

During murine macrophage apoptosis induced by co-incubation of *T. vaginalis* for 8 h, there was downregulation of Bcl-xL, an anti-apoptotic protein. Downregulation of Bcl-xL expression is associated with caspase-3 activation, as caspase-3 is involved in the downregulation of Bcl-xL. On the other hand, the overexpression of Bcl-2 did not protect macrophages from apoptosis nor affected cytochrome c release or Bax and caspase-3 activation [35]. The release of cytochrome c by mitochondria activated the caspase-3, which, in turn, activated the p38 MAPK pathway and resulted in host cell death [36]. Another way for *T. vaginalis* to induce macrophage cell death is via pyroptosis due to NLRP3 inflammasome activation. This activation needs two signals: the priming step and the activation step. In the priming step, NF-kB is activated and consequently the genes encoding pro-IL1β and NLRP3 increased transcription levels. The activation step is initiated by the host cells danger-associated molecular patterns (DAMPS), such as ATP, and pathogen-associated molecular patterns (PAMPs), such as bacterial surface proteins. Human macrophages in contact with the parasite lead to NLRP3 inflammasome activation via P2X7 receptor, which interacts with extracellular ATP. Once activated, there is an increase in capase-1 activity, leading to bioactive IL-1β generation through the processing of pro-IL-1β. As a consequence of inflammasome activation, macrophages are killed by pyroptotic cell death [37].

Finally, immune system cells also have their ways of preventing the establishment of *T. vaginalis* infection. Neutrophils have two ways to eliminate pathogens: phagocytosis or neutrophil extracellular traps (NET). In NET, a rearrangement of the cytoskeleton occurs, by which nuclear DNA is released into the extracellular DNA. Thus NET pathogens are entrapped and killed. The death caused by NET is known as NETosis. In mice, trichomonads stimulated neutrophils to produce NETs via ROS generation and ERK1/2 and p38 MAPK signaling pathways [38]. After the *in vitro* co-incubation of *T. vaginalis* and human neutrophils under NET-forming conditions, the parasite grew 35.2%. In addition, 32.9% of trophozoites remained intact after this contact. This data showed the involvement of NETs, as suggested by [39]. In vaginal discharges, NETs may have different phenotypes with *T. vaginalis* stimulation [40]. Nevertheless, a few years earlier, the death of the parasite was shown to be independent of NETosis. The contact between PMNs and *T. vaginalis* was necessary to kill the parasite via trogocytosis, a killing mechanism by which neutrophils “nibble on” pathogens. The participation of serine peptidases and components present in human serum were important in this process. A pre-treatment with AEBSF, a serine peptidase inhibitor, decreased parasite death by more than 85%. Meanwhile, in the absence of human serum, protozoan death by neutrophils decreased, suggesting that opsonization took a part in trogocytosis [41]. Likewise, human monocytic cells used extracellular traps (ET) to capture *T. vaginalis* using the same pathway and formation as proposed above. Furthermore, in this context, both live and dead parasites could induce ET formation [42].

**Figure 1:**
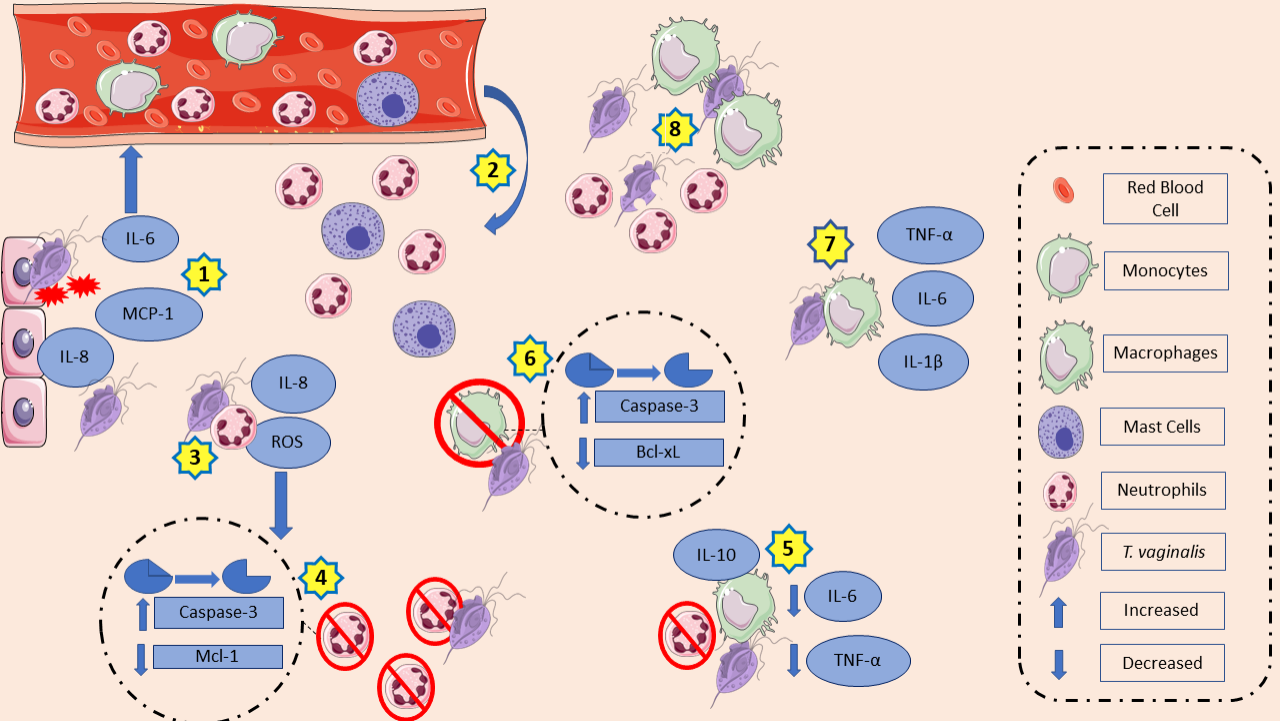
FIGURE 1: *T. vaginalis* and the interaction with host immune cells. *T. vaginalis* stimulates vaginal, ectocervical, and endocervical epithelial cells to release IL-6, MCP-1 and IL-8 (1), resulting in the migration of mast cells and neutrophils to the site of infection (2). There, neutrophils in contact with trichomonads release IL-8 and ROS (3), which activate caspase-3 and decrease Mcl-1, an anti-apoptotic protein (4). Macrophages in contact with neutrophils killed by parasite-induced apoptosis decrease IL-6 and TNF-α and increase IL-10 (5). The interaction between macrophages and *T. vaginalis* can lead to their apoptosis by caspase-3 activation and downregulation of Bcl-xL (6) or can result in the release of TNF-α, IL-6, and IL-1β (7). Finally, monocytes can kill the protozoan through extracellular traps, while neutrophils use a mechanism known as trogocytosis (8).

*T. vaginalis* has been shown to stimulate vaginal, ectocervical, and endocervical epithelial cells to release cytokines that can recruit and activate innate immune cells such as neutrophils, macrophages, and mast cells of the infection site. At the infection site, the parasite has a complex machinery that modulates ROS, cytokine, and chemokine production and induces apoptosis of the host's immune cells. It also interferes in the maturation of antigen-presenting cells, such as dendritic cells. All together, these mechanisms allow pathogen survival and infection establishment. **Fig. 1** summarizes the main mechanisms discussed in this topic.

## WAR CONSEQUENCES: ASSOCIATION TO HIV, OUTCOMES DURING PREGNANCY, AND PROSTATE CANCER

The presence of *T. vaginalis* has been associated with several clinical complications such as prostate cancer, preterm birth, and increased acquisition and transmission of HIV [43]. Due to the importance of this subject, this section focuses on the main immunological mechanisms described in the last two decades, exceptionally the paragraph “HIV association,” which included articles outside this time period due to its high relevance.

Infection with *T. vaginalis* increased the risk of acquiring HIV by 1.5 times compared to uninfected individuals. In addition, women with HIV showed a trichomoniasis prevalence of 17-20%, with high rates of repeated infection (22.7% in about 16 months) [44, 45]. Some mechanisms may explain this association. It is suggested that *T. vaginalis* promotes an alteration in the vaginal microbiota by reducing lactobacilli producers of H_2_O_2_, causing a change in pH and favoring BV. In turn, BV enhanced pro-inflammatory cytokines that disrupted the mucosal barrier, while trichomonads disrupt the integrity of the cell monolayer by themselves. Both mechanisms may facilitate HIV-1 passage. The immune response activation due to the *T. vaginalis* infection observed in *in vitro* studies is expected to lead to recruitment of CD4 lymphocytes, the target cells of HIV. Peripheral blood mononuclear cells (PBMCs) infected with HIV-1 in contact with the parasite released TNF-α, which is partially involved with enhancing viral replication [46–49].

Women with positive results for *T.* vaginalis-antigen test showed lower levels of secretory leukocyte protease inhibitor (SLPI). *In vitro*, the parasite's cysteine peptidases cleave the SLPI, a defense mechanism cited against acquisition of HIV infection [50]. However, a cross-sectional study found no association between the presence of *T. vaginalis* and the enhanced expression or activation of CD4+ cells of the HLADR+CD38+ phenotypes, which is in disagreement with the aforementioned studies that suggested it as one of the pathways involved in facilitating HIV infection [51].

In pregnant women, the parasite leads to low birth weight and preterm delivery. The molecular mechanisms for this association are still to be identified [52]. In a population of pregnant women with less than 20 weeks of gestation, co-infection with *T. vaginalis* and BV increased IL-1β and IL-8. It has been suggested that high levels of IL-1 β in response to colonization of anaerobic Gram-negative bacteria may possibly be associated with preterm delivery between 18-22 weeks of gestation [53, 54]. In agreement with these findings, a cohort study evaluated 65 asymptomatic pregnant patients infected with *T. vaginalis*. The concentration of cervical IL-8 and neutrophil defensins in vaginal fluid were higher in women with asymptomatic trichomoniasis than in the uninfected group, confirming cytokine release and neutrophil activation during trichomoniasis [55]. Furthermore, *T. vaginalis* infection during pregnancy increased the serum concentration of granulocyte-macrophage colony-stimulating factor (GM-CSF). GM-CSF is predicted to stimulate the production of immune cells such as macrophages, dendritic cells, and granulocytes. Serum C-reactive protein (CRP) levels were also high in *T. vaginalis*-infected women. Both findings indicate a systemic inflammatory response [56].

The symbiosis between *T. vaginalis* and *M. hominis* may play a role in the clinical worsening observed in trichomoniasis during pregnancy. *In vitro* studies have found that after treatment with metronidazole, large amounts of bacteria were released in the extracellular environment. Once they are free in this environment, the bacteria can infect human cells [57]. Another study found a prevalence of 9.5% of this bacterial microorganism. It is cited as one of the causes of intra-amniotic infection, which may result in preterm birth [58].

The association between *T. vaginalis* and prostate cancer is debatable. While some studies have shown this mechanism to be involved in signaling tumor-like cells, other studies have not found epidemiological evidence [59, 60]. Due to the focus of this review, the topic below will discuss the immunological mechanisms described for this association. Epithelial mesenchymal transition (EMT) is the transformation of epithelial cells into stromal cells, contributing to tumor progression. In this context, human prostate epithelial cells co-incubated with live *T. vaginalis* increased CCL2 mRNA, but not its protein levels, while CXCL8, IL-1β, and IL-6 increased both protein and mRNA levels. This interaction induced EMT via NF-kB and JAK/STAT signal pathway. Meanwhile, an increase in H_2_O_2_ generation via the NADPH oxidase system has suggested an involvement in the regulation of ERK, while NF-kB inhibitors decreased IL-1β production, suggesting a role of NF-kB in IL-1β production. Some proposed that IL-1β and CXCL8 can lead to monocyte migration. IL-6, via the IL-6R/JAK2/STAT3 signaling pathway, stimulated polarization of M2 macrophages, which accumulate in prostate tumor tissue and contribute to the proliferation of prostate cancer cells [61–63]. *In vivo*, prostate cancer cells from PC3 lineage cells with *T. vaginalis*-contained medium (TCM) injected into a PC3 xenograft mouse model resulted in an increase in tumor weight and volume and in EMT-related markers, proliferating cell nuclear antigen (PCNA) and cyclin D1, which are proliferative signal molecules [64]. Interestingly, adipocytes can also increase prostate cancer development. Co-incubation of mouse prostate cancer cells with *T. vaginalis* made adipocytes to increase production of IL-4, CCL2, and IL-6, as well as mRNA levels of CCL2, IL-4, and IL-13. In turn, IL-4 and IL-13 stimulated macrophage migration and polarization to M2 macrophages [65]. Prostate stromal cells infected with trichomonads recruited neutrophils and monocytes by CXCL8, CCL2 released via activation of TLR4, ROS, MAPK, and NF-kB [66]. Benign prostatic hyperplasia (BPH) cells stimulated by the parasite can in turn stimulate proliferation of prostate stromal cells. BPH cells in contact with trichomonads (TCM) produced cytokines and chemokines (CXCL8, CCL2, IL-1β, and IL-6), which led to mast and monocytes cell migration and activated mast cells. In turn, prostate stromal cells in contact with activated mast cells stimulated by the TCM supernatant (M-TCM) increased CXCL8 and CCL2 levels. These cytokines seem to be involved in prostate stromal cell proliferation via CXCL8-CXCR1 and CCL2-CCR2 signaling. Furthermore, prostate stromal cells showed an increase in cyclin D1 and B-cell lymphoma-2 (Bcl-2) expression in response to incubation with supernatant of M-TCM [67, 68]. BPH cells stimulated with *T. vaginalis* were subsequently incubated with mast cells. This supernatant was then put in contact with prostate stromal cells. The result was that this supernatant stimulated BPH epithelial cells proliferation. IL-6 seems to be involved in the proliferation of these cells and in EMT induction [69]. Mouse cells showed the same behavior. *In vitro*, media collected from mouse prostate cancer (PCa) cells in contact with *T. vaginalis* stimulated an increased migratory ability of macrophages. This was expected to occur through the increased production of CCL2 and CXCL1 detected in co-incubation media. Macrophages exposed to PCa *T. vaginalis* media showed an increased expression of CCL2, IL-6, and TNF-α at mRNA level. Conditioned media from *T. vaginalis*-infected PCa cells incubated with macrophages stimulated PCa cell proliferation and invasiveness. Furthermore, these cells expressed the cytokine receptors GP130, CCR2, and CXCR2, expected to be positively involved with the proliferation and invasiveness of these cells [70]. *In vivo*, when the mouse prostate was injected with a mixture of trichomonads and PCa cells, there was an increase in cyclin D1 and N-cadherin expression, opposed to a decrease in E-cadherin expression. In addition, this group showed a larger volume and weight of prostate cancer compared to the group injected only with PCa cells [71].

Finally, TvMIF production by *T. vaginalis* possibly contributes to an inflammatory environment through the interaction with monocytes and CD74 receptors, the HuMIF receptor. TvMIF binds to the CD74 receptor and leads to downstream ERK1/2, Akt, and BAD activation involved with increased cell proliferation and inflammation [72]. Interestingly, TvMIF is frequently found in patients with positive rather than negative infection, suggesting there is a TvMIF release in the extracellular environment *in vivo*. In addition, it has been detected especially in men, either because of the stronger immune response of men against TvMIF or because there is a greater release by the parasite in men than in women. TvMIF release possibly contributes to increased risk of human prostate cancer due to increased cell proliferation and invasion [72].

The long permanence of *T. vaginalis* may cause several clinical complications. Three of them are highlighted: the increase in HIV transmission and acquisition due to the disruption of the mucosal barrier, reduction of SLPI, and recruitment of CD4^+^T cells; adverse pregnancy outcomes caused by a systemic inflammatory response with cytokine production, neutrophil migration, and *M. hominis* release into the extracellular environment; and prostate cancer attributed to a sequence of inflammatory mechanisms that increased the invasiveness and growth of prostate cells, creating a perfect microenvironment for the establishment of cancer.

## AN EXTRA HELP OF *M. HOMINIS* AGAINST HOST IMMUNE RESPONSE

The endosymbiosis between *M. hominis* and *T. vaginalis* has gained prominence in recent years. Some articles showed that the bacteria can survive within the parasite's cytoplasmic vacuoles and their presence can influence *T. vaginalis* biochemical pathways and immunomodulation. It is predicted to contribute to tumorigenesis and adverse pregnancy outcomes. Thus, this association may affect the immune response, survival, and outcomes observed during trichomoniasis [73]

This interaction increased the mycoplasma drug resistance and protected *M. hominis* from antibiotics. The 5-Bromodeoxyuridine (5-BrdU) incorporation assay suggested that the bacterial DNA is incorporated into trichomonad DNA. The assay is based on the lack of mitochondria in the parasite and therefore absence of extranuclear DNA. Thus, 5-BrdU incorporation in a location different than the nucleus indicates replication activity from another organism. Cultures of *T. vaginalis* with *M. hominis* were submitted to 5-BrdU assay. As a result, 5-BrdU incorporation was detected, which indicated DNA synthesis by the bacteria inside the parasite. The bacteria identity was confirmed using anti-*M. hominis* antibodies. Mycoplasma DNA incorporation into *T. vaginalis* DNA could possibly affect PCR-based analytical methods, a golden pattern of trichomoniasis laboratorial diagnosis [74–76]. On the other hand, the effects on trichomonads' metronidazole resistance are under debate. Some authors argue that *T. vaginalis* resistance to metronidazole cannot be attributed to the presence of *M. hominis* [77–80], while others speculate that bacterial presence plays a role in modulating enzymatic expression levels related to resistance, such as pyruvate ferredoxin oxidoreductase (PFOR), and demonstrates that seven of eight resistant isolates were *M. hominis*-positive [81, 82]. Furthermore, it is possible that this endosymbiosis may actually reduce resistance to metronidazole, making the isolate more susceptible and suggesting that downregulation of PFOR is not necessarily related to drug resistance [83].

The difference among *T. vaginalis* isolates appears to be related with the number of infecting bacteria, although this endosymbiosis was not observed in all *T. vaginalis* isolates [84]. Interestingly, *in vitro Mycoplasma*-positive *T. vaginalis* isolates can infect both *Mycoplasma*-free *T. vaginalis* isolates and human cells, while a single *T. vaginalis* isolate can be infected by two mycoplasma species [83, 84]. Recent studies have shown that *T. vaginalis* can be infected not only by *M. hominis*, but also by *Candidatus Mycoplasma girerdii*, previously known as Mnola, which showed a 78% similarity with *M. hominis* and 85% with *Mycoplasma genitalium.* In addition, the hypothesis that the protozoan may have symbiosis with other bacteria, such as *Ureaplasma* spp., needs further explanation [85, 86]. In the presence of any *Mycoplasma* species, the parasite showed an upregulation of hemolysis and cytoadherence in epithelial cells, indicating a bacterial influence on the virulence observed in *T. vaginalis* isolates [87].

*M. hominis* and *T. vaginalis* also affect the immune response. The presence of both microorganisms in symbiosis stimulated IL-8, TNF-α, IL-1β, and IL-23 production by human macrophages and activated the NF-kB pathway, which seems be involved in this cytokine production [88]. In addition, the bacteria may influence the growth rate of *T. vaginalis*, being ∼20% faster in the presence of the symbiont. Furthermore, a competition between macrophages and bacteria for arginine-free uptake leads to downregulation of NO production by macrophages [89]. Nevertheless, this combination potentiated the cytopathic effects on epithelial cells with low viability and increased in the intercellular spaces, suggesting an increase in virulence since there was a higher rate of amoeboid transformation and phagocytic activity. However, there was no impact on the leukotoxic effects caused by *T. vaginalis* [90, 91]. Interestingly, PBMCs in contact with small extracellular vesicles from isolates containing *T. vaginalis* virus inhibited the IL-8 response to the signaling of *Mycoplasma*-derived macrophage-activating lipopeptide-2 (MALP-2), a mycoplasma protein, thus reducing the immune surveillance against bacteria [92].

*T. vaginalis* and *Mycoplasma* species have a beneficial relationship for both parties. For the bacteria, the parasite works as a “trojan horse,” with extra protection against immune defense and antibiotics in the extracellular environment; for the parasite, there is an increase in growth rate, virulence, and modulation of immune response, allowing its survival under stress conditions. Further studies are needed on the possibility of *T. vaginalis* carrying other microorganisms, such as *Ureaplasma* spp.

## IMMUNOMODULATORY WEAPONS: *T. VAGINALIS* AND PURINERGIC SIGNALING AND CD59 SEQUESTRATION

*T. vaginalis* has enzymes known as ecto-nucleoside triphosphate diphosphohydrolase (E-NTPDase) and ecto-5'-nucleotidase (E-5N). They are responsible for a cascade that hydrolyzes extracellular nucleotides from tri-, di-, and monophosphate nucleotides to the nucleosides, such as ATP, ADP, and AMP to adenosine (ADO). Extracellular nucleosides not only affect the infection establishment, but also are essential for parasite survival since *T. vaginalis* cannot synthetize *de novo* purine and pyrimidine rings, requiring an uptake from the host via salvage pathway. ADO showed anti-inflammatory properties, which are important in the context of establishing an infection. However, studies have shown that some *T. vaginalis* isolates are deficient in AMP hydrolysis activity [93]. During human infection, this may result in leukocyte infiltration due to low adenosine levels, leading to the acute symptoms observed in patients infected with those isolates [93]. Furthermore, adenosine interacts and activates A2A receptors present in immune cells such as neutrophils, macrophages, and monocytes. Meanwhile, ATP is released during cell damage, plays a role in cytotoxic mechanisms, and stimulates chemotaxis of neutrophils and mast cells, as well as increases cytokines with proinflammatory properties [94]. Interestingly, adenosine nucleotides are preferable for uptake by the parasite than guanosine [95]. Under serum restriction conditions, adenosine deaminase enzymatic activity does not change, while E-5'N had an increased gene expression; it acts as a compensatory pathway, increasing adenosine levels. Under low iron availability conditions, adenosine deaminase decreases its activity, which returns to normal conditions when there is exposure to iron sources [96, 97]. Nucleotides also influence virulence factors. A very cytotoxic isolate had its injurious effects potentiated in the presence of ATP accumulation, but when adenosine deaminase, responsible for the conversion of adenosine into inosine, was inhibited by EHNA, the increased availability of extracellular adenosine protected cells against these effects [98]. These findings shed light on the potential of inhibiting ecto-nucleotidases. Lycorine and candimine, alkaloids from Amaryllidaceae plants, were tested as compounds targeting *T. vaginalis* enzymes. There was an inhibition of NTPDase and ecto-5'-nucleotidase activities after 24 h, suggesting a new way to affect the parasite-host interaction. Lycorine also increased ROS production by neutrophils, which can be an adjuvant in the clearance process against the parasite [99, 100].

In addition to the aforementioned enzymes, *T. vaginalis* can also use the sequestration of CD59 from different cells, including erythrocytes, as an immune evasion strategy. CD59 is a human protectin that acts as a complement lysis-restricting factor. Therefore, its sequestration partially increases the parasite protection against complement-mediated lysis [101].

In conclusion, NTPDases, E-5'N, and adenosine deaminase provide the parasites with enzymatic mechanisms to modulate the inflammatory environment, contributing to parasite survival. In the same context, the sequestration of CD59 is also one of the ways to evade immune response, helping to avoid targeting by the complement system.

## HOPE IN THE MIDST OF WAR: ANTIBODY PRODUCTION DURING TRICHOMONIASIS

During *T. vaginalis* infection, there is antibody production. Although they are not protective against reinfection, they may be associated with the presence of symptoms or the parasite itself. In this context, some authors have tried to find an association between certain antibodies and symptoms.

Using the mouse model, a study evaluated the circulating antibodies during infection caused by *T. vaginalis*. IgA was present in vaginal washes and serum in greater levels in mice infected with *T. vaginalis* isolates from asymptomatic patients. Also, IL-2, CD3^+^ T cells, CD4^+^ T cells, and NK cells were also with higher levels in mice infected with *T. vaginalis* isolates from asymptomatic vs. symptomatic patients [102]. Fourteen days after infection, IgG, IgG1, and IgM levels were higher in serum samples and vaginal washes of rodents infected with isolates from symptomatic patients than in isolates from asymptomatic rodents [103]. In agreement, when antibodies in the serum of symptomatic patients were analyzed, IgG and IgM were detected in 66.6% and 33.3% of patients, respectively (400 dilutions). IgA was detected in 100% of cases (>400 dilutions). In vaginal washes, IgM was not detected in symptomatic patients, while IgG was detected in all patients tested (≥40 dilutions) [104].

Antibody production is also associated with protection to adverse outcomes of pregnancy (AOP) and therapy success. Pregnant women positive for *T. vaginalis* with no AOP had a higher IgG anti-TvLG in vaginal secretion than the group with AOP [105]. After effective therapy with metronidazole, a low circulating IgG anti-*T. vaginalis* was detected, suggesting that there was no longer antigenic stimulation. However, in few patients whose treatment failed, reinfection or infection by resistant isolates led to the detection of antibody, which can be associated with a chronic infection [106].

In conclusion, IgG was found in serum samples of rodents infected with isolates from symptomatic patients and patients with no symptoms. However, antibody production is not likely protective. The association between symptoms and the presence of antibodies remains under investigation.

## THE INFINITY STONES: VACCINE CANDIDATE TARGETS

Some proteins of *T. vaginalis* play an essential role in the establishment of infection and in the parasite-host relationship. They are, therefore, promising vaccine candidate targets.

Cysteine protease (CP) 30 was found in all 20 fresh isolates from symptomatic and 20 asymptomatic patients investigated in India. The intensity band of CP30 and the basal cytoadherence to vaginal epithelial cells were higher in isolates from symptomatic than from asymptomatic patients. Although the antibodies anti-CP30 inhibited cytoadherence in asymptomatic and symptomatic isolates, the specific inhibition of cytoadherence was lower in asymptomatic isolates vs. symptomatic ones. Thus, CP30 was suggested as a virulence factor of *T. vaginalis* [107]. The group of BALB/c mice immunized with intranasal injection of 62 kDa cysteine-proteinase (p-62) plus cholera toxin (CT) increased IgG1, IgG2a, and IgG3 serum circulating levels. In sera of the immunized group of p-62 plus CpG-oligodeoxynucleotides (CpG), there was an increase in IgG2a, IgG2b and IgG3. Vaginal secretion in both groups showed IgG and IgA levels specific for p-62. However, IgA response was higher in p-62 plus CT group [108]. A few years later, *T. vaginalis* anti-protease monoclonal antibodies (MAbs) named 4D8 and 1A8 were tested against this same protease. The result showed that the antibodies reacted with different epitopes of a repetitive nature. After treatments with pronase, heat and trichloroacetic acid (TCA), reactivity decreased due the epitope alteration, affecting MAbs recognition. In conclusion, 4D8 obtained a better protective activity in murine model than 1A8, which can be explained by the interaction of MAbs with different protein epitopes [109]. *In vitro*, 4D8 and 1A8 also inhibited cytoadherence to HeLa cells. 62kDa anti-protease MAbs, in the presence of macrophages, increased NO levels. However, there was no effect on cysteine protease of *T. vaginalis* antigen [110].

Two peptides from α-actinin present in the parasite (ACT-F and ACT-T) were used at high subcutaneous dosages for immunization. ACT-T conferred a protection of 100% to mice, while the high dosage of ACT-F only protected 55% of the animals. High amounts of IFN-γ, IL-17A and IL-6 cytokines and barely any IL-2 and IL-4 were detected in both tested groups. In addition, both peptides produced high levels of IgG, specifically IgG1, which was detected at greater concentrations than IgG2 [111]. The transient receptor potential-like channel of *T. vaginalis* (TvTRPV) was also tested as a vaccine candidate target. At high doses, there was an increased immune response in BALB/c mice with high IgG production in serum. In addition, TvTRPV can be recognized by macrophages, which stimulated the production of IL-1β, IL-6 and TNF-α. CD4^+^T cells isolated from TvTRPV-immunized mice were incubated with mouse macrophages treated with TvTRPV. As a result, CD4^+^T cells produced IL-10 and IFN-γ [112]. The latter is expected to potentiate macrophage cytotoxicity against the protozoan, while IL-10 is associated with the initial anti-inflammatory feedback loop [112].

A unique N-terminus from enolases, a group of enzymes that participates in glycolysis, was also suggested as a potential target against *T. vaginalis*. However, in this study, there was no development or test as an immunizing agent [113]. Finally, whole *T. vaginalis* cells were proposed as an immunization strategy. The mice group that received the vaccine candidate had higher IgG and IgG1 serum total levels than the unimmunized group. The production of IgG2a varied between the groups tested [49]. Similarly, the pretreatment of mice with TvDNA two days before the parasite inoculation resulted in less available trophozoites in vaginal washes and infection signs. Higher levels of IL-6 were observed between four to 14 days post-infection in the pre-treated group compared to the untreated group. There was a higher production of IL-17 throughout the 14 days after infection. However, there were undetectable levels of IL-10 from four to ten days after the infection [114].

One of the proposed ways to fight trichomoniasis is immunization. However, due to lack of an adequate animal model for *T. vaginalis* infection, this becomes an arduous task. Animal models that are used to study trichomoniasis infection present some important limitations. The route of inoculation can be subcutaneous, intraperitoneal, or vaginal. However, the first two are not the site of *T. vaginalis* infection in the host, therefore they do not accurately mimic the infection in humans. In the intraperitoneal route, successful infection is associated with gross-pathologic changes, ascitic fluid, and mortality [115, 116]. Another way to infect mice is through vaginal infection. This model is useful to understand the virulence of the parasite. However, for the infection to occur and be sustained, it is necessary that animals are pretreated with subcutaneous estradiol and intraperitoneal dexamethasone. An analysis of the presence of PMNs indicated that this treatment inhibited the migration of these cells, which are expected to affect the immunological analysis of this animal model [115, 117]. Furthermore, pretreatment with bacteria was also tested. A pre-inoculation of mice with *Lactobacillus acidophilus* increased *T. vaginalis* infection duration. Interestingly, there was no alteration in the vaginal microbiome of mice after *Lactobacillus* inoculation. However, prior to *L. acidophilus* inoculation, a subcutaneous estradiol injection is required. Treatment with estrogen may increase the vaginal antibody response [115, 118], which affects the accuracy of antibody analysis, important in vaccine development studies.

**Table 1** summarizes the vaccine candidate targets and the immune system activation discussed in this topic.

**Table 1.** Vaccine candidate targets against trichomoniasis.

Vaccine candidate targets	Antibody response	Cytokine response	Ref.
Cysteine Protease 30 (CP30)	Not mentioned	Not mentioned	[107]
62 kDa protease + cholera toxin	IgG1, IgG2a, IgG3 (sera); IgA and IgG (vaginal secretion)	Not mentioned	[108]
62 kDa protease + CpG-oligodeoxynucleotides	IgG2a, IgG2b and IgG3 (sera); IgA and IgG (vaginal secretion)	Not mentioned	[108]
ACT-T	IgG; (IgG1 > IgG2)	IFN-γ, IL-17A, IL-6 and IL-10	[111]
ACT-F	IgG; (IgG1 > IgG2)	IFN-γ, IL-17A, IL-6 and IL-10	[111]
TvTRPV	IgG	IL-1β, IL-6, TNF-α, IL-10 and IFN-γ	[112]
N-terminal from enolases	Not mentioned	Not mentioned	[113]
T. vaginalis whole cells	IgG and IgG1. Variable IgG2a	Not mentioned	[49]
TvDNA	Not mentioned	IL-6 and IL-17	[114]

## CONCLUSION

This review highlights parasite strategies to activate and stimulate or evade variated and complex immunological mechanisms related to the symptoms and clinical complications observed here. The symbiosis with *M. hominis* also requires further studies to understand the extent of this relationship. As a conclusion of this review, the war between *T. vaginalis* and the host immune system is far from over. Further studies in immunology are necessary to understand the pathogen behavior aiming to discover new targets to fight this infection.

## Abbreviations:

AOP: – adverse outcomes of pregnancy;
BMDC: – bone marrow-derived dendritic cell;
BPH: – benign prostatic hyperplasia;
BV: – bacterial vaginosis;
CP: – cysteine proteases;
CT: – cholera toxin;
EMT: – epithelial mesenchymal transition;
ET: – extracellular trap;
HuMIF: – human macrophage migration inhibitory factor;
iTreg: – inducible regulatory T cell;
LG: – lipoglycan;
LPG: – lipophosphoglycan;
MAb: – monoclonal antibody;
NET: – neutrophil ET;
NO: – nitric oxide;
PBMC: – peripheral blood mononuclear cell;
PCa: – prostate cancer;
PMN: – polymorphonuclear cell;
RNI: – reactive nitrogen intermediate;
ROS: – reactive oxygen species;
SLPI: – secretory leukocyte protease inhibitor;
STI: – sexually transmitted infection;
TCM: – T. vaginalis containing medium;
TvLG: – T. vaginalis LG;
TvMIF: – T. vaginalis macrophage migration inhibitory factor;
TvSP: – T. vaginalis secretory product;
TvTRPV: – transient receptor potential-like channel of T. vaginalis;
VEC: – vaginal epithelial cell.
